# The Influence of Climatic Factors on the Provocation of Epileptic Seizures

**DOI:** 10.3390/jcm13123404

**Published:** 2024-06-11

**Authors:** Thilo Hammen, Sebastian Treib, Philipp Treib, Hermann Stefan, Hajo M. Hamer, Ralf Landwehr, Lynn Lohmann, Sebastian Koch, Johannes Treib, Werner Adler

**Affiliations:** 1 Clinic for Neurology, Friedrich-Alexander-University Hospital Erlangen, 91054 Erlangen, Germany; hermann.stefan@t-online.de (H.S.); hajo.hamer@uk-erlangen.de (H.M.H.); 2 Clinic for Neurology, Westpfalz-Klinikum Kaiserslautern, 67655 Kaiserslautern, Germany; rlandwehr@westpfalz-klinikum.de (R.L.); jtreib@westpfalz-klinikum.de (J.T.); 3 Clinic for Neurology, University Hospital Homburg, 66421 Homburg, Germany; sebastiantreib1904@gmail.com (S.T.); lohmannlynn@gmail.com (L.L.); 4 Department of Periodontology and Operative Dentistry, University Medical Center of the Johannes Gutenberg University Mainz, 55131 Mainz, Germany; philipptreib@gmail.com; 5 Clinical Neurology, Miller School of Medicine, University of Miami, Coral Gables, FL 33146, USA; skoch@med.miami.edu; 6 Department of Biometry and Epidemiology, Friedrich-Alexander-University Erlangen, 91054 Erlangen, Germany; werner.adler@fau.de

**Keywords:** climatic factors, seasonal variation, trigger factors, seizures, epilepsy

## Abstract

**Background/Objectives:** Recent studies provide the first indications of the impact of climate factors on human health, especially with individuals already grappling with internal and neurological conditions being particularly vulnerable. In the face of escalating climate change, our research delves into the specific influence of a spectrum of climatic factors and seasonal variations on the hospital admissions of patients receiving treatment for epileptic seizures at our clinic in Kaiserslautern. **Methods:** Our study encompassed data from 9366 epilepsy patients who were admitted to hospital due to epileptic seizures. We considered seven climate parameters that Germany’s National Meteorological Service made available. We employed the Kruskal–Wallis test to examine the correlation between the frequency of admittance to our hospital in the mentioned patient group and seasons. Furthermore, we used conditional Poisson regression and distributed lag linear models (DLMs) to scrutinize the coherence of the frequency of patient admittance and the investigated climate parameters. The mentioned parameters were also analyzed in a subgroup analysis regarding the gender and age of patients and the classification of seizures according to ILAE 2017. **Results:** Our results demonstrate that climatic factors, such as precipitation and air pressure, can increase the frequency of hospital admissions for seizures in patients with general-onset epilepsy. In contrast, patients with focal seizures are less prone to climatic changes. Consequently, admittance to the hospital for seizures is less affected by climatic factors in the latter patient group. **Conclusions:** The present study demonstrated that climatic factors are possible trigger factors for the provocation of seizures, particularly in patients with generalized seizures. This was determined indirectly by analyzing the frequency of seizure-related emergency admissions and their relation to prevailing climate factors. Our study is consistent with other studies showing that climate factors, such as cerebral infarcts or cerebral hemorrhages, influence patients’ health.

## 1. Introduction

It is already known that different internal and external triggers can provoke epileptic seizures. These include psychological stress, sleep deprivation, sensory stimulation, unreliable medication, alcohol withdrawal, or hyperventilation [1,2,3,4].

Due to a fast-progressing climate change and the resulting aggravating climatic factors and weather conditions, we decided to investigate the influence of climatic factors on the frequency of emergency admissions of epilepsy patients due to seizure therapy.

In our study, we correlated multiple climatic factors in the hospital area of our clinic, which is placed in a moderate climatic zone (271 m above standard altitude zero height) in Southwest Germany to the number of admissions of patients who were treated for epileptic seizures in the emergency unit and subsequently further diagnosed and treated during the inpatient stay in our clinic for Neurology.

Seizures were classified according to the instruction manual for the ILAE 2017 operational classification of seizure types described in Fisher et al. [5].

## 2. Material and Methods

### 2.1. Patient Recruitment

All epilepsy patients who were admitted from 1 January 2001 to 31 December 2020 to our clinic in Kaiserslautern in an emergency due to epileptic seizures were included in our study.

An emergency medical history was taken, and the patients were admitted to the Department of Neurology for the following days. During the inpatient stay, experienced neurology specialists classified the patient’s seizure type. Medical history and seizure semiology were ascertained by taking the patient’s personal and external anamnesis, including that of a third party like an emergency doctor or relatives of the patient. In addition, further diagnostics included at least a high-resolution cranial MRI imaging and EEG examinations.

We must note at this point that we did not record the exact frequency of epileptic seizures of individual patients during the period of admission to our clinic.

In our study, only the frequencies of emergency admissions due to epileptic seizures in different weather conditions were analyzed and correlated with the regional climate factors investigated.

Nevertheless, it can be assumed in this context that the frequency and severity of the seizures had escalated at the time of admission to the emergency department due to a temporary acute worsening of the seizure situation.

To ensure the anonymity of patient data, every patient received an anonymous identification number. According to the ILAE 2017 Classification system, seizures were classified into 4 groups according to their seizure onset: seizures with focal onset, seizures with generalized onset, seizures with unknown onset, or seizures that remained unclassified due to inadequate information or inability to be placed into other categories.

The patients with focal epilepsy in our study had the underlying structural lesions typical of this patient group, such as cerebral infarctions, cerebral hemorrhages, or injuries caused by severe traumatic brain injuries, as well as cavernomas, sclerosis, or congenital malformations of the cerebral cortex.

We investigated the frequency of admissions to our emergency unit due to epileptic seizures regarding year, months, seasons of the year, and weekdays. The number of admittance seizures per day was modeled by a weather parameter and adjusted by year, month, and weekday using conditional Poisson regression [6]. Distributed lag linear models (DLMs) [7] were used to examine a time-delayed effect of the weather on the frequency of seizures, with a lag of 0 to 7 days.

Due to these models, an overall effect in this time period was calculated, and the incidence rate ratio (IRR) was stated.

### 2.2. Measurement of the Weather Data

We considered six different climate parameters that Germany’s National Meteorological Service, located in Essen, made available.

The following weather data were acquired:

We chose Celsius [°C] as the unit for temperature and hectopascal [hPA] as the unit for air pressure.

- Daily maximum temperature: daily maximum temperature 2 m above ground [°C].
- Daily mean temperature: daily mean temperature 2 m above ground [°C].
- Daily minimum temperature: daily minimum temperature 2 m above ground [°C].
- Humidity: daily average of relative air humidity [%].
- Precipitation: daily total precipitation, measurement time 06:50 pm [1/qm].
- Air pressure: daily average of air pressure, hospital area Westpfalz-Klinikum Kaiserslautern (271 m above NHN).
- A seventh parameter was calculated by ourselves to cover the within-day variability of the temperature in one single parameter: daily difference in temperature (difference between maximum and minimum temperature).

### 2.3. Statistics

Using Kruskal–Wallis tests, we investigated whether there was any connection between the frequency of seizures and the year’s seasons, weekdays, months, and years. Thus, we could decide whether to consider seasonal trends in addition to weather conditions in further analysis. We used conditional Poisson regression and distributed lag linear models (DLMs) to analyze the association between climate parameters and the frequency of admissions of patients to our emergency unit due to epileptic seizures, corrected for year, month, and day of the week. As a period of time, we used eight days with a lag of 0–7 days. This is because the possible influence of weather parameters might impact patients’ admissions to the emergency unit a couple of days later. We calculated the overall effect and indicated it as an incidence rate ratio (IRR).

Our results are presented in tables or forest plots. For statistical evaluation, we used the program R V4.1.3 (R Core Team, Vienna, Austria, 2022). The level of significance was *p* = 0.05.

## 3. Results

### 3.1. Number of Patient Admissions Divided into Individual Subgroups and Seizure Classifications According to the ILAE 2027

From 1 January 2001 to 31 December 2020, 9366 epilepsy patients were admitted to the emergency department to receive treatment for epileptic seizures, as mentioned above. A total of 117 patients had multiple diagnoses, of which the most current one was evaluated. We observed 5048 male and 4318 female patients with an average age of 45.2 years and a standard deviation of 28.1 years. Patient admissions divided into the seizure classification show that 3062 admissions were due to focal seizures with impaired awareness, including motor- and non-motor onset and possible propagation from focal to bilateral tonic-clonic seizures. In total, there were 2200 admissions due to seizures with unknown onset, 1459 due to seizures with generalized onset including motor- and non-motor seizures, 788 due to seizures that remained unclassified, and 605 patients’ admissions due to seizures with focal onset aware including motor- and non-motor onset and possible propagation from focal to bilateral tonic-clonic seizures.

### 3.2. Investigation of the Seasonal Influence

In our period of observation, the average number of patients admitted to our emergency unit due to epileptic seizures registered a day was 1.28. Here, seizures classified as seizures with focal onset in patients with impaired awareness occurred most frequently (*n* = 3062), and seizures with focal onset in aware patients occurred in (*n* = 605), respectively. Seizures with generalized onset (including motor and non-motor (absence) occurred in (*n* = 1459); seizures with unknown onset occurred in (*n* = 2200); and seizures that remained unclassified occurred in (*n* = 788), respectively.

The lowest number of patient admissions to our emergency unit due to epileptic seizures was in 2018, with 413 admissions. However, the highest number, 546, was in 2008. This is a significant difference in the number of epilepsy patient admissions due to seizure occurrence between the mentioned years (Kruskal–Wallis test: *p* < 0.001).

The month with the highest frequency of patient admissions due to seizures a day was October, with 1.41 admissions a day. The second-highest frequency of admissions was in January, with 1.36 admissions a day. In June, the frequency of admissions was the lowest, with 1.14 admissions a day.

**Figure 1:**
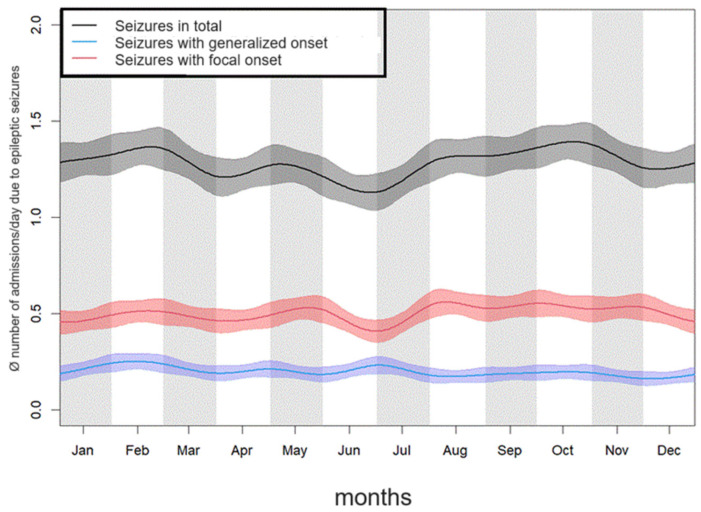
Average frequency of emergency admissions to hospital of patients due to epileptic seizures in the individual months over the course of the year.

The difference in the frequency of patient admissions to the emergency room between the months showed significant differences in the overall population (Kruskal-Wallis test: *p* = 0.001). However, this was only the case in the subgroup analysis of patient admissions due to generalized seizures (*p* = 0.012), but not for focal seizures (*p* = 0.179). The results are shown in Table 1 and Figure 1.

Our study showed that the number of patient admissions due to epileptic seizures was significantly higher in fall and winter compared to spring and summer (Kruskal–Wallis-Test: *p* = 0.005). This relates to global patient admissions due to epilepsy and in patients admissions whose seizures were classified with focal onset and impaired awareness as well as in seizures with unknown onset. The corresponding results are shown in Table 2.

When analyzing the frequency of patient admissions on the individual days of the week, our study found that significantly fewer patients visited the emergency department due to epileptic seizures on weekends (Sat. and Su.) than on weekdays (Kruskal–Wallis test: *p* < 0.001). This was true for the total number of epilepsy patients and the respective seizure types determined in subgroup analysis. Results are demonstrated in Table 3. 

### 3.3. Investigation of the Climatic Influence

All models examining the relationship between weather parameters took into account 8 days with lag 0 to 7 and were corrected for factors that showed significant seasonal influence, as described in Section 3.2. Therefore, our description of the results is somewhat simplified to increase the readability of important findings.

In the overall patient population, increased mean daily temperatures showed a significant reduction in the number of patients admitted to the emergency unit due to epileptic seizures.

(IRR = 0.990, *p* = 0.038), while increased humidity significantly increased the frequency of patient admissions to our emergency unit (IRR = 1.005, *p* = 0.043).

**Figure 2:**
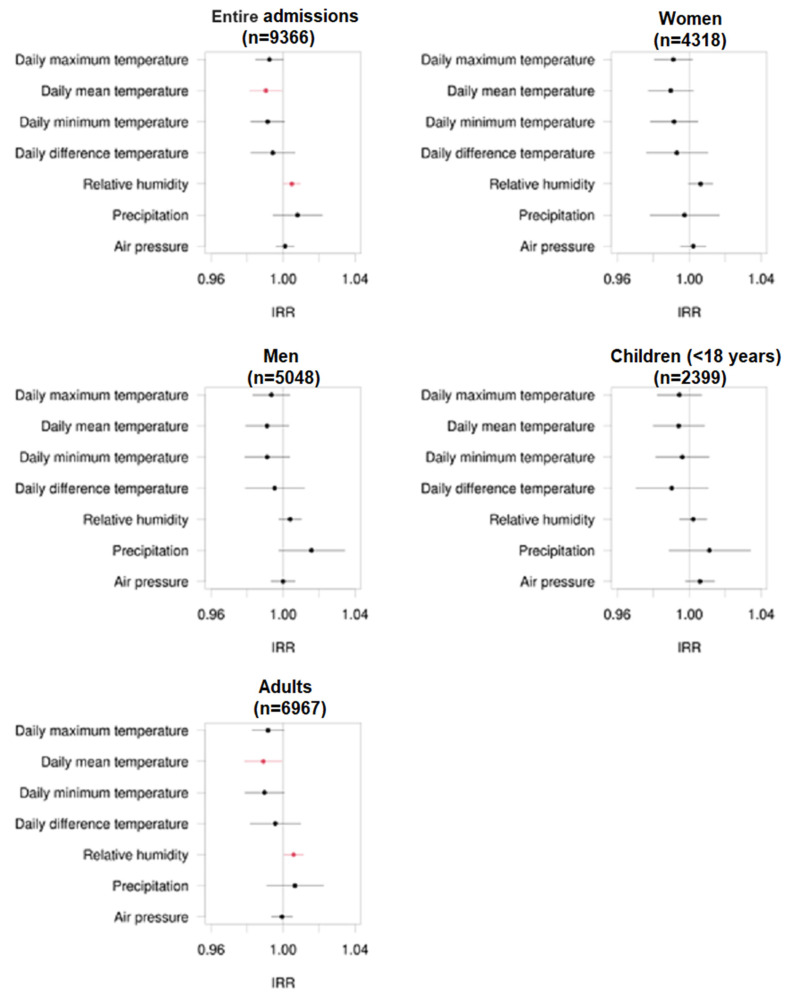
Forest plots regarding the incidence rate ratio (IRR) of admissions of epilepsy patients to the emergency unit due to epileptic seizures in the entire patient group and different subgroups of women, men, children, and adults and corresponding different climate factors. The numbers of patient admissions are displayed in brackets. The black dots represent non-significant correlations, while the red dots represent significant correlations.

Comparable results were seen in the adult patient group ≥ 18 years. Again, increased daily temperatures were associated with a reduced frequency of admission to the hospital. In contrast, increased humidity was associated with a significant increase in admission to the hospital based on seizures. In patients ≤ 18 years of age, none of the climate parameters studied significantly affected emergency admission to the hospital. Similarly, no significant differences were seen when comparing male and female patients in this context. Mentioned results are displayed using forest plots of the mentioned subgroups in Figure 2.

Comparing different seizure types of patients admitted to the emergency unit due to seizures, our study showed that climate factors influenced the frequency and the propensity of admissions of epilepsy patients with generalized seizure onset, including motor and non-motor (absence) seizures, and in patients whose seizures were classified as seizures with unknown onset or whose seizures remained unclassified.

**Figure 3:**
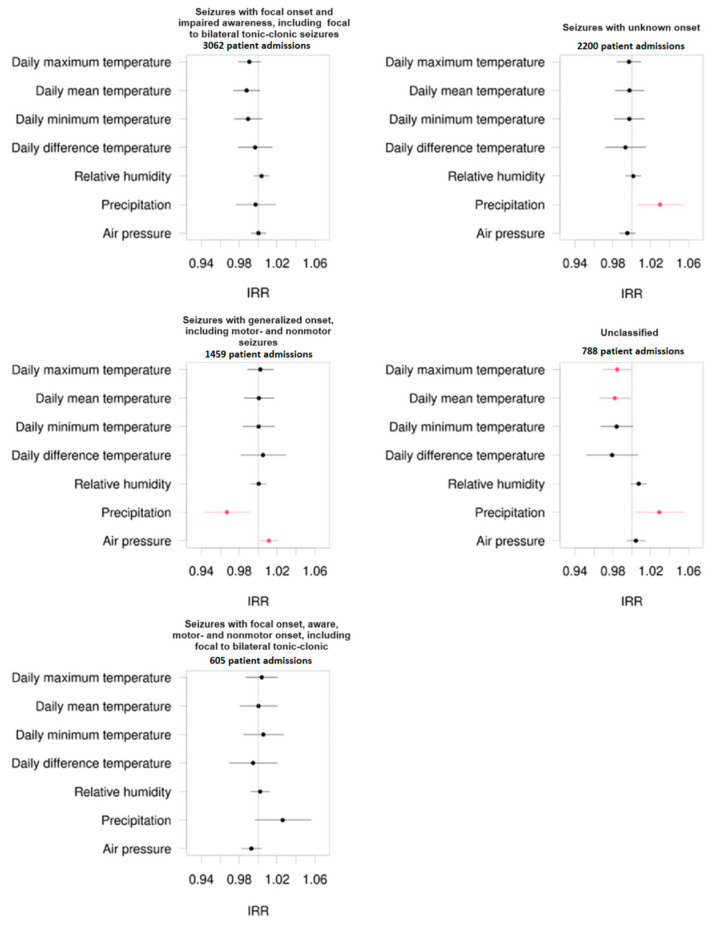
Forest plots regarding the incidence rate ratio (IRR) of admissions of epilepsy patients to the emergency unit divided into different seizure types classified by the ILAE 2017 and corresponding to different climate factors. The numbers of patient admissions are displayed in brackets. The black dots represent non-significant correlations, while the red dots represent significant correlations.

As demonstrated in Figure 3, precipitation has a significant effect on the frequency of admission in patients whose seizures were classified as seizures with generalized onset, seizures with unknown seizure onset, or unclassified seizures.

In this context, high precipitation protects patients from admission to the emergency unit based on seizures in patients with generalized epilepsy (IRR = 0.967, *p* = 0.008). On the other hand, high precipitation levels promoted the frequency of admission in patients whose seizures were classified as seizures with unknown seizure onset (IRR = 1.030, *p* = 0.022) or unclassified seizures (IRR = 1.029, *p* = 0.025).

Further connections were detected between hospital admission frequency and air pressure in patients with generalized seizures (IRR = 1.011, *p* = 0.018). Here, increased air pressure correlated with an increased frequency of admissions to the emergency unit due to seizures in the mentioned patient group.

The hospital admission frequency of patients with unclassified seizures significantly correlates to the daily maximum temperature (IRR = 0.984, *p* = 0.039) and the mean average temperature (IRR = 0.982, *p* = 0.032). Hospital admission frequency in patients with seizures with unknown onset also significantly correlates to the mean average temperature (IRR = 1.022, *p* = 0.026).

Mentioned results are displayed using forest plots of the mentioned subgroups in Figure 3.

**Figure 4:**
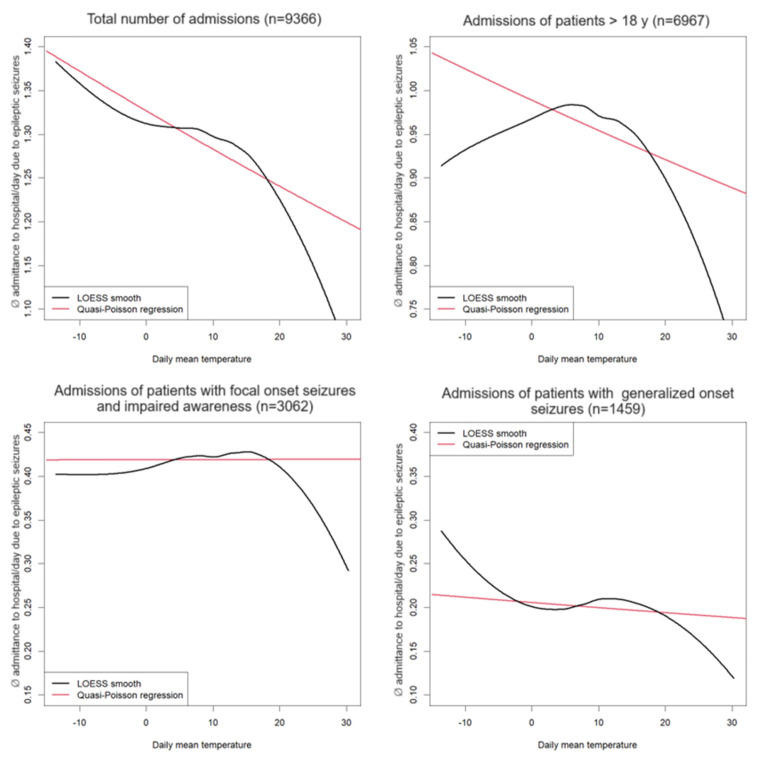
Correlation between daily temperature and admittance to the emergency room per day due to epileptic seizures in the entire patient group and subdivided among their corresponding seizures classified according to ILAE 2017. The total number of patient admissions is displayed in brackets. Non-linear local polynomial regression (black) compared to quasi-poisson regression with linear dependency structure (red).

Figure 4 shows a smoothed relationship between daily admittance to the hospital based on seizures and daily mean temperature using local polynomial regression (LOESS). This relationship is compared to uncorrected quasi-poisson regression with daily mean temperature as independent and daily admittance to the hospital based on seizures as dependent variables, where the influence of temperature is linear. Diagrams for the total population and subgroups of adult patients, seizures with focal onset with impaired awareness, and seizures with generalized onset are shown.

The figure shows that the influence is relatively linear for the total number of patients and the number of patients ≥ 18 years (i.e., there are no strong directional changes in the curves, although this is no longer so clear in adult patients).

However, although there can be seen a dependency of admittance to hospital from daily mean temperature in patients suffering from seizures with focal onset with impaired awareness and patients suffering from seizures with generalized onset by non-local polynomial regression, no clear (or a very weak) linear relationship is identified by quasi-poisson regression here.

## 4. Discussion

Although there are currently only a limited number of clinical studies investigating the influence of climate factors as possible triggers of seizures in epilepsy patients, there is growing evidence that climate-related stress factors influence the frequency of seizures in the mentioned patient group.

Our study revealed that precipitation and air pressure significantly influenced the admission rate of epilepsy patients to our clinic for emergency treatment due to seizures.

In our study, patients with generalized seizure onset, precipitation, and air pressure significantly affected seizure activity. In the mentioned patient group, higher air pressure increased the frequency of emergency admissions of epilepsy patients due to seizures to our hospital.

On the other hand, high precipitation was significantly correlated with reduced patient admissions to the emergency unit based on seizures in the mentioned patient group.

In contrast, the frequency of admissions in patients who suffer from seizures with focal onset was not significantly influenced by mentioned or other climate factors in our study.

Brás et al. and Rakers et al. also discovered a significant correlation between atmospheric pressure and seizure risk, which increased by 14%, with a lag time of one day, for every 10.7 hPa decrease in barometric pressure. In patients with less severe epilepsy, who were treated with only one antiepileptic drug, the risk of seizures even increased by 36%. In a non-linear model, the risk of seizures decreased by up to 26% at air pressures above 980 hPa. An air pressure of less than 980 hPa increased the risk of seizures by 29%. Both results were obtained after a lag time of one day [3,8].

In summary, the results of studies dealing with climate factors as possible trigger factors of seizures are heterogeneous. The results show different findings when comparing changes in individual climate parameters and the rate of patient admissions to the hospital due to seizures.

Table 4 provides an overview of studies dealing with climate factors and their impact on the clinical course of seizures in epilepsy patients.

Comparing seasonal influence, our study demonstrated that significantly more epilepsy patients were treated for seizures in our emergency department in the fall and winter. The most frequent admissions occurred in October, with January being the second most frequent. The admission frequency of epilepsy patients due to seizures was lowest in June.

This was the case for epilepsy patients with generalized seizures. Patients admitted due to focal seizures showed no significant differences in the monthly distribution of seizure-related admissions in terms of admission frequency.

The studies by Bras et al., Chiang et al., Alexandratou et al., and Clemens et al. [8,9,10,11] listed in Table 4, also demonstrated a seasonal influence on the development of epileptic seizures.

The results of the mentioned studies, except those of Alexandratou et al. [9] are comparable to ours. Bras et al. [8] recorded an increased uptake of epilepsy patients in winter. The months of January and February were the months with the highest admissions in the study by Chiang et al. [10] In the study by Clemens et al. [11], the most frequent admissions due to epileptic seizures were recorded in January. The frequency of admissions then fell continuously and reached its lowest level in August, only to rise again continuously until January.

On the other hand, in the study by Alexandratou et al. [9], June was identified as the month with the highest frequency of patient admissions.

Looking at the patient admissions on the respective days of the week, we found that there are significantly fewer admissions of epilepsy patients to the hospital due to seizures on weekends. This may be because epilepsy patients are more likely to live alone at the weekends, or care capacity is reduced at the weekends, particularly in nursing homes. This may result in a low rate of emergency calls by relatives or nursing staff at weekends compared to weekdays.

In our study’s overall patient population, increased mean daily temperatures significantly reduced the number of patients admitted to the emergency unit due to epileptic seizures. In contrast, increased humidity significantly increased the frequency of patient admissions to our emergency unit.

The studies by Jacks et al., Chang et al., Bras et al., Ruegg et al., and Rakers et al. also confirmed the influence of ambient temperature on the frequency of admissions due to epileptic seizures in epilepsy patients [3,8,14,15,16].

While Chang et al. and Bras et al. [8,16] showed that a drop in daytime temperature led to a significant increase in seizure-related hospital admissions, the studies by Ruegg et al. and Rakers et al. showed that high ambient temperatures led to a decrease in admissions.

Our study only considers linear influences of the individual climate parameters, which means nonlinear influences are not captured by the models. To investigate whether there might be a nonlinear influence, we examined the relationship between daily seizures and daily mean temperature through visual inspection of nonlinear local polynomial regression curves (LOESS smooth), compared to uncorrected quasi-poisson regression where the influence of daily mean temperature is linear.

The results of our study show that the influence is relatively linear for the total number of patients, especially in the patient group of over 18 years. This means that there are no strong directional changes in the curves.

However, although a dependency of seizures from daily mean temperature in patients suffering from seizures with focal onset with impaired awareness and patients suffering from seizures with generalized onset was seen with non-local polynomial regression, no clear (or a very weak) linear relationship was identified via quasi-poisson regression in this point.

In any case, this could explain the differences between our findings and the results in the literature.

Further possible reasons for different results in this context are that seasonal influence can vary in different parts of the world. For example, winters are more brutal or milder in certain regions. Even so, summer temperatures are moderate in northern countries. In contrast, heat waves in summer in Mediterranean countries represent a more stressful and, therefore, have a higher impact as a significant event as a trigger factor for seizures in epilepsy patients.

In addition, a change in climatic and weather factors is taking place in some regions in a shorter period of time with an increased extent of extreme weather conditions.

This may be a greater trigger factor for the development of epileptic seizures than moderate fluctuations in weather or climate over a longer period of time.

Furthermore, an evaluation of seizure frequency may contain sources of error, primarily if the seizure frequency is based on data from seizure calendars that are unreliably kept or in case seizures are not observed or amnestied by epilepsy patients.

Another reason for the different results of the studies dealing with a possible connection between epileptic seizures and climate factors may be because the studies to date either do not classify the patients’ seizures or use different classification systems for epilepsies.

This may result in different or non-significant results comparing individual climate parameters as trigger factors for different seizure types of epilepsy patients.

A central key point of our study findings is that climate factors essentially did not affect focal epilepsies. In contrast, seizures of patients with generalized epilepsy are prone to be triggered by the latter.

In essence, our study demonstrated that climatic factors, such as precipitation and air pressure, significantly influence seizure frequency in patients with generalized epilepsies, which are based on a genetic background in most cases. In contrast, climatic factors do not significantly influence the clinical course of focal, most likely structurally metabolic epilepsies, which are based on a genetic background only in a few cases.

The final clarification of the underlying genetic pathophysiology has not been conclusively elucidated today. Studies mentioned in the following in Table 5 support the results of our research that the seizure course of generalized genetic epilepsies is more prone to climate factors than focal epilepsies, mainly based on structural-metabolic lesions.

Gulcebi et al. review potential associations between climatic factors and the disposition of possible seizure triggering in genetic epilepsies [28].

The authors discuss possible explanations that seizures in patients with genetic epilepsy are more prone to be triggered by climatic factors based on individual human genetic variants that lead to altered physiological responses to temperature by reducing the thermoregulatory capacity of affected patients.

Studies give strong evidence that seizures in some genetic epilepsy syndromes are sensitive to fever. This implies that the probability of seizure provocation by elevated body temperature is significantly higher in the affected patient group than in epilepsy patients who do not show a genetic disposition [29].

For example, patients suffering from Dravet syndrome demonstrating prolonged febrile convulsions at the onset of epilepsy, even mild elevations in body temperature due to fever or increased ambient heat result in severe seizure series often resistant to therapy [21]. The mentioned disease is associated with pathological variants in the gene SCN1A, which encodes the temperature-sensitive ion channel NaV1.1 [30].

Evidence that genetic generalized epilepsies are more susceptible to climatic factors is also confirmed through the rat model for genetic epilepsies (Strasbourg Absence Epilepsy Rats (GAERS)), which represents a well-validated rodent model for absence epilepsies [31]. Although the rats mentioned are derived from a single, genetically homogeneous colony in Strasbourg sharing precisely the same genetic mutation in the Cacna1h gene, different spike-wave discharges in the EEG represent an increased disposition to epileptic seizures, as well as different seizure phenotypes and behavioral traits varied between GAERS colonies in the respective institutes in Melbourne, Strasbourg, Istanbul, and Grenoble [27].

Most likely, different climatic factors and environmental conditions at each institute led to the mentioned expression of epileptic discharges, and different seizure frequencies and intensities in the genetically identical rat strains.

Studies whose results strongly indicate an increased seizure disposition triggered by climatic factors in epilepsies based on a genetic background are briefly summarized in Table 5.

Our study demonstrated, as mentioned above, that patients with focal epilepsies, most of which are not genetically determined, are more resistant to climatic factors regarding seizure provocation factors than genetic, mostly generalized epilepsies. This fact is also confirmed in animal models mentioned in the following.

Electrically induced kindling is an established animal model for focal, structural-metabolic epilepsy. In the mentioned model, the mentioned method reduces the seizure threshold in a circumscribed focal brain area [32,33].

Animal studies in which focal epilepsy was provoked in rats via the above methods showed that seasonal influences had no significant effect on seizure activity [34,35].

To our knowledge, only one British clinical study, mentioned in Table 4, has demonstrated that sunlight has a seizure-suppressing effect in patients with focal epilepsy [19]. This study showed that the frequency of focal seizures with impaired consciousness was significantly reduced on sunny compared to cloudy days.

In addition to the aforementioned direct influences of climate factors on seizure triggering, climate change could also indirectly influence the disease course of epilepsy patients by amplifying possible further indirect trigger factors of epileptic seizures.

In this context, the current climate change could increase stress, especially in critical climate zones where heat periods are unbearable. Increased stress levels are mainly based on social and emotional stress caused by, among other things, a limited supply of food and drinking water.

Studies by Bartolini et al. and Ferlisi et al. demonstrated that emotional stress significantly triggers seizures in epilepsy patients, most likely due to epilepsy-promoting factors like stress hormones [36,37]. The studies of den Heier et al. demonstrated that elevated cortisol levels correlate positively with interictal EEG discharges, representing increased neuronal excitability. On the other hand, elevated cortisol levels correlate negatively with global functional connectivity in the EEG [38].

Furthermore, climate change leads to a continuous significant increase in average and extreme temperatures during the day, especially in night phases with frequent tropical nights even in Central Europe. This results in a reduced proportion of regenerating deep sleep phases, resulting in a lasting negative effect on sleep architecture [39,40,41,42]. The resulting sleep deficit indirectly leads to an increased susceptibility to seizures, especially in patients with genetic epilepsies [37].

We have to note at this point that in our study, we did not assess patients’ sleep deprivation by questionnaires prior to admission to our clinic and, therefore, cannot assess with ultimate certainty this factor in the development of seizures. The same applies to seizures provoked by unreliable medication intake, psychosocial stress, or alcohol withdrawal. These aspects of possible seizure triggering were also not explicitly recorded in our study. However, it should be noted at this point that the trigger factors for seizures mentioned are randomly distributed among all patients. Therefore, all patients in the study are largely equally affected by the mentioned trigger factors, which consequently do not significantly influence the results in our study concerning the effect of investigated climatic factors.

However, it should be noted at this point that sleep deprivation as a trigger factor is randomly distributed normally among all patients. Therefore, all patients in the study are largely equally affected, and sleep deprivation does not significantly influence seizures in our study for that reason.

Another approach to explain the interaction between climate factors and increased seizure frequency is based on indirect influences of climate factors and the altered pharmacology of anti-seizure medication (ASM). In this context, it is assumed that the efficacy of ASMs differs under different climatic conditions due to their stability or pharmacokinetics being negatively affected by higher external temperature or altered humidity. Some authors suggest that seasonal variation in ASM efficacy is correlated to reduced drug levels based on higher excretion through sweat during hot weather in terms of increased patients’ perspiration [43,44,45].

Another aspect in this context is probably based on ASM’s unfavorable storage conditions, which are increasingly exposed to sunlight, heat, and increased humidity due to climate change. The impaired stability of pharmaceuticals results in reduced efficacy and bioavailability of the ASM, thereby promoting seizures in the patient. This has been demonstrated in particular for ASM like carbamazepine, phenytoin, valproate, and phenobarbital preparations, for which bioavailability was reduced by up to 50% under the conditions mentioned above [46,47].

## 5. Conclusions

As climate change intensifies meteorological and climatic factors and, consequently, their impact on human health in the following decades, it is urgently necessary to expand research on the effects of altered climate parameters and their impact on the course of diseases in the future.

Multicenter, continent-related clinical studies and basic science studies, such as animal studies, are crucial in reliably assessing and comparing the influence of different global climatic factors on the genetic and pathophysiological basis of epilepsy.

Genetic dispositions hold a key position regarding the impact of climate factors on seizure frequency at this time. Therefore, genetic aspects should also be the focus of future studies on the relationship between climate factors and health.

In future planned studies, the collected parameters should be compared using a joint study design, as recently mentioned by the ILAE/AES Translational Task Force [48,49].

## Figures and Tables

**Table 1:** Total number and average frequency of hospital admissions per day (in brackets) of all seizures and seizures classified by ILAE 2017 in different months of the year. Significance (*p*) calculated using Kruskal–Wallis test.

Months	1	2	3	4	5	6	7	8	9	10	11	12	*p*
Entire admissions	845 (1.36)	749 (1.33)	800 (1.29)	741 (1.24)	789 (1.27)	681 (1.14)	740 (1.19)	815 (1.31)	796 (1.33)	877 (1.41)	785 (1.31)	749 (1.21)	0.001
Seizures with focal onset with impaired awareness	261 (0.42)	227 (0.4)	251 (0.4)	239 (0.4)	264 (0.43)	219 (0.36)	236 (0.38)	274 (0.44)	289 (0.48)	283 (0.46)	266 (0.44)	253 (0.41)	0.206
Seizures with unknown onset	217 (0.35)	177 (0.31)	181 (0.29)	158 (0.26)	178 (0.29)	134 (0.22)	172 (0.28)	193 (0.31)	195 (0.32)	231 (0.37)	183 (0.3)	181 (0.29)	0.002
Seizures with generalized onset	146 (0.24)	143 (0.25)	128 (0.21)	123 (0.2)	122 (0.2)	128 (0.21)	119 (0.19)	122 (0.2)	105 (0.17)	126 (0.2)	108 (0.18)	89 (0.14)	0.012
Unclassified seizures	58 (0.09)	74 (0.13)	69 (0.11)	70 (0.12)	66 (0.11)	60 (0.1)	74 (0.12)	68 (0.11)	64 (0.11)	56 (0.09)	65 (0.11)	64 (0.1)	0.903
Seizures with focal onset, aware	51 (0.08)	46 (0.08)	48 (0.08)	52 (0.09)	57 (0.09)	55 (0.09)	49 (0.08)	60 (0.1)	43 (0.07)	51 (0.08)	54 (0.09)	39 (0.06)	0.798

**Table 2:** Total and average number per day (added in brackets) of epilepsy patients admitted to the hospital due to seizures. In the second row, all patient admissions are displayed. In rows 3–7, patients’ admissions are divided according to classifications of underlying seizures.

Seasons	Spring	Summer	Fall	Winter	*p* (Kruskal–Wallis)
Entire admissions	2330 (1.27)	2236 (1.22)	2458 (1.35)	2342 (1.3)	0.005
Seizures with focal onset with impaired awareness	754 (0.41)	729 (0.4)	838 (0.46)	741 (0.41)	0.032
Seizures with unknown onset	517 (0.28)	499 (0.27)	609 (0.33)	575 (0.32)	0.005
Seizures with generalized onset	373 (0.20)	369 (0.20)	339 (0.19)	378 (0.21)	0.586
Unclassified seizures	205 (0.11)	202 (0.11)	185 (0.10)	196 (0.11)	0.742
Seizures with focal onset, aware	157 (0.09)	164 (0.09)	148 (0.08)	136 (0.08)	0.589

**Table 3:** Total and average number of epilepsy patients admitted to hospital per day (added in brackets) due to seizures in total and divided into underlying seizures classified according to ILAE 2017 on corresponding days of the week.

Weekdays	Mon	Tue	Wed	Thu	Fri	Sat	Sun	*p* (Kruskal–Wallis)
Entire admissions	1567 (1.5)	1589 (1.52)	1554 (1.49)	1432 (1.37)	1220 (1.17)	1008 (0.97)	996 (0.95)	<0.001
Seizures with focal onset with impaired awareness	510 (0.49)	493 (0.47)	525 (0.5)	433 (0.41)	405 (0.39)	366 (0.35)	330 (0.32)	<0.001
Seizures with unknown onset	360 (0.34)	374 (0.36)	341 (0.33)	339 (0.32)	302 (0.29)	244 (0.23)	240 (0.23)	<0.001
Seizures with generalized onset	260 (0.25)	284 (0.27)	219 (0.21)	232 (0.22)	187 (0.18)	127 (0.12)	150 (0.14)	<0.001
Unclassified seizures	131 (0.13)	164 (0.16)	156 (0.15)	107 (0.1)	86 (0.08)	70 (0.07)	74 (0.07)	<0.001
Seizures with focal onset, aware	97 (0.09)	98 (0.09)	107 (0.1)	102 (0.1)	74 (0.07)	64 (0.06)	63 (0.06)	0.001

**Table 4:** Overview of clinical studies concerning influence of climate factors on seizure activity.

Authors	Topic of Studies and Results
**Studies demonstrating a significant effect of seasonal influence on seizure activity**	
Brás et al. 2018	Analysis of 377 seizure episodes of adult epilepsy patients in a hospital in Lisabon. The authors demonstrated that a significantly higher incidence of seizures was found in winter [8].
Alexandratou et al., 2020	In total, 1351 epileptic seizures of 143 adult patients were treated in an outpatient epilepsy clinic in Athens, Greece. In the study, partial-onset seizures developed a peak in June [9].
Chiang et al., 2020	Hospital visits due to epileptic seizures were significantly higher in January and February compared to March to December [10].
Clemens et al., 2013	The study included 45,833 seizures based on the seizure diaries of 62 epilepsy patients. Average seizure counts peaked in January (9.7%) and declined through August (7.4%). After that, the monthly number of seizures gradually increased until December (9.6%). The difference between the percentage averages in August and January was 31.1%, demonstrating a significant seasonal effect [11].
**Studies demonstrating no significant effect of seasonal influence on seizure activity** (Altimiras-Roset et al., 2014; Asensi et al., 1977; Rüegg et al., 2008) [12,13,14]	
**Studies concerning the impact of temperature on seizure activity**	
Jacksch, 2018	A significant reduction in seizures is associated with extensive temperature fluctuations [15].
Chang et al., 2019	The authors found that for every 1 °C drop in temperature, the number of emergency visits for epilepsy increased by 1.6%. Temperatures below 18 °C had the highest predictive value for seizures [16].
Brás et al., 2018	The authors discovered a statistically significant relationship between lower ambient temperatures and increased seizures. However, this was only true when two or more seizures occurred [8].
Rakers et al., 2017; Rüegg et al., 2008	High ambient temperatures demonstrate a significant reduction of epileptic seizures and status epilepticus. Rakers et al. demonstrated that temperature above 20 °C significantly reduced the risk of seizures in 604 adult patients by 46% in the entire study population with a lag time of 1 day [3,14].
Altimiras-Roset et al., 2014	Altimiras-Roset et al. could not establish a statistical relationship between temperature and seizure frequency. The average temperature on days with at least one seizure was 12.4 °C, slightly lower than on seizure-free days with 12.9 °C [12].
**Studies concerning the impact of air pressure on seizure activity**	
Brás et al., 2018; Rakers et al., 2017	The authors discovered an almost linear negative correlation between atmospheric pressure and seizure risk, which increased by 14%, with a lag time of one day, for every 10.7 hPa decrease in barometric pressure. In patients with less severe epilepsy, who were treated with only one antiepileptic drug, the risk of seizures even increased by 36%. In a non-linear model, the risk of seizures decreased by up to 26% at air pressures above 980 hPa. An air pressure of less than 980 hPa increased the risk of seizures by 29%. Both results were obtained after a lag time of one day [3,8].
**Studies demonstrating no significant effect of air pressure on seizure activity** (Altimiras-Roset et al., 2014; Asensi et al., 1977; Boldrey and Millichap, 1966; Chang et al., 2019; Doherty et al., 2009; Jacksch, 2018) [12,13,15,16,17,18]	
**Studies concerning the impact of humidity on seizure activity**	
Brás et al., 2018; Chiang et al., 2020; Rakers et al., 2017	High relative humidity above 80% increased the risk of seizures by up to 48% in the entire study population 3 days after exposure. For every 18.4% increase in relative humidity, the risk of seizures increases by 22% in the overall study population and by 47% in patients younger than 60 years with a lag time of 3 days [3,8,10].
**Studies demonstrating no significant effect of humidity on seizure activity** (Altimiras-Roset et al., 2014; Baxendale, 2009; Chang et al., 2019) [12,16,19]	
**Studies concerning the impact of daily sunshine duration on seizure activity**	
Baxendale, 2009; Brás et al., 2018	While Brás et al. discovered a statistically significantly higher incidence of seizures on days with shorter daylight hours, Baxendale et al. demonstrated a significant negative correlation between sunshine duration and the number of focal seizures with impaired consciousness. In the study, seizures were less likely on bright, sunny days and more common on cloudy, overcast days [8,19].
**Studies demonstrating no significant effect of total coverage but a clear tendency (*p*-value = 0.07) of sunshine duration on seizure activity** (Chang et al., 2019) [16]	

**Table 5:** Overview of studies demonstrating that temperature is a main trigger factor in genetic epilepsies.

Authors	Topic of Studies and Results
Mei et al., 2019; Verbeek et al., 2015	Patients with Dravet syndrome (DS) with pathogenic SCN1A mutations were compared with those of a cohort with childhood epilepsy and a community-based cohort with epilepsy. In 99% of patients with DS, the parents recalled at least one seizure trigger. Seizure precipitants reported in more than half of the cohort with cDS were fever (97%), cold (68%), bathing (61%), acute moments of stress (58%), and physical activity (56%). The study highlighted elevated body temperature as an important seizure precipitant in patients with DS, whether due to fever, warm bath, ambient heat, or physical exertion [20,21].
Griffin et al., 2016; Menezes and Da Silva, 2017	Graffin et al. demonstrated that zebrafish’s unique characteristics, in combination with genetic alterations, are further changing our understanding of epilepsy and helping to identify personalized therapeutics for specific patient cohorts. The authors used Pentylenetetrazol (PTZ) to induce a seizure-like state in zebrafish to study the basic mechanisms of epilepsy. In the work of Menez et al., the influence of sex, weight, and temperature changes on the latency of adult zebrafish to reach classical seizure states induced by PTZ was investigated. Sex and weight did not affect the response to the PTZ profile. When the water temperature was changed from 22 to 30 °C, the lower temperature prolonged the latency to reach seizure states, and the higher temperature significantly decreased, demonstrating increased seizure susceptibility under increased temperature compared to the control group maintained at 26 °C. In the mentioned study, blockade of kainate receptors by DNQX (10μM) did not prevent the increased susceptibility of adult zebrafish exposed to hyperthermia and PTZ-induced seizures. NMDA blocked by MK-801 (2.5 μM) prevented the additive effect of hyperthermia on PTZ effects in adult zebrafish. This result emphasizes that water temperature in the PTZ model in adult zebrafish is a confounding factor, as it can directly affect the response to PTZ, especially through a mechanism related to NMDA receptors [22,23].
Hunt et al., 2012	The authors described an in vivo model of hyperthermia-induced seizures in zebrafish larvae at 3 to 7 days post-fertilization. Bath-controlled temperature changes are rapid and reversible in this model. Acute electrographic seizures after transient hyperthermia showed age dependence, exercise independence, and no mortality. Electrographic seizures recorded in the forebrain of larval zebrafish were blocked by the addition of antagonists to the transient receptor potential vanilloid channel (TRPV4) or to the N-methyl-d-aspartate (NMDA) glutamate receptor to the bathing medium. In conclusion, our results suggest a role of heat activation of TRPV4 channels and enhanced NMDA receptor-mediated glutamatergic transmission in hyperthermia-induced seizures [24].
Sun et al., 2012; Wang et al., 2004	The work of Sun et al. reported that the knock-in of a GEFS+ SCN1A mutation (K1270T) into the Drosophila sodium channel gene, para, caused a semi-dominant temperature-induced seizure phenotype. Electrophysiological studies of GABAergic interneurons in the brain of adult GEFS+ flies reveal a novel cellular mechanism underlying heat-induced seizures: the deactivation threshold for persistent sodium currents reversibly shifts to a more negative voltage when the temperature increases. Furthermore, the data of the study suggest that a natural temperature-dependent shift in sodium current deactivation (exacerbated by mutation) may contribute to febrile convulsions in GEFS+ and possibly in healthy individuals. Wang et al. described a novel paralytic gene mutant, the Nubian mutant, identified in a behavioral screen for conditionally temperature-sensitive seizure mutants in Drosophila melanogaster. Nubic mutants exhibit altered synaptic structure and defective neurotransmitter release by disrupting phosphoglycerate kinase (PGK), an enzyme required for ATP formation in the final stage of the glycolytic pathway. Alterations in ATP metabolism likely disrupt similar signaling pathways in humans, as PGK deficiency is associated with mental retardation, seizures, and exercise intolerance. Given the behavioral similarities between disruptions in PGK function in Drosophila and humans, analysis of nubic animals may reveal conserved neuronal responses associated with altered ATP formation in the brain. Disruption of ATP formation in Nubian animals is associated with temperature-dependent defects in neuronal activity, with initial seizure activity accompanied by activity-dependent loss of synaptic transmission [25,26].
Powell et al., 2014	The Genetic Absence Epilepsy Rats from Strasbourg (GAERS) represent the most widely used animal model of genetic epilepsy. The authors compared seizure, behavioral, and brain morphometry characteristics of four main GAERS colonies under active international study: two from Melbourne (MELB and STRAS-MELB), one from Grenoble (GREN), and one from Istanbul (ISTAN). The authors demonstrated that seizure characteristics varied between colonies, with MELB GAERS exhibiting the least severe epilepsy phenotype in terms of seizure frequency and GREN GAERS exhibiting four times more seizures than MELB. A previously identified mutation in the Cacna1h gene, which controls the CaV 3.2 T-type calcium channel (R1584P), was present in all four GAERS colonies but was absent in all non-epileptic control rats [27].

## Data Availability

Data are contained within the article.
